# The photooxidative sensitization of bis(*p*-substituted diphenyl)iodonium salts in the radical polymerization of acrylates

**DOI:** 10.1039/c9ra05413h

**Published:** 2019-09-10

**Authors:** Alicja Balcerak, Janina Kabatc

**Affiliations:** UTP University of Science and Technology, Faculty of Chemical Technology and Engineering Seminaryjna 3 85-326 Bydgoszcz Poland nina@utp.edu.pl +48 52 374 9009 +48 52 374 9112

## Abstract

The ability of two-component dyeing photoinitiating systems for the radical polymerization of 1,6-hexanediol diacrylate (HDDA) and 2-ethyl-2-(hydroxymethyl)-1,3-propanediol triacrylate (TMPTA) is presented. The systems under study comprised a hemicyanine dye as a sensitizer and iodonium salts that played a role of a coinitiator. The kinetic parameters of the polymerization reaction, such as the rate of polymerization (*R*_p_) and the degree of conversion of monomer (*C*_%_), were estimated. The thermodynamic feasibility of an electron transfer process in the systems studied was verified and calculated using the Rehm–Weller equation. It was found that a benzoxazole derivative in the presence of iodonium salts effectively initiated the polymerization of acrylate monomers. The polymerization rates of about 10^−7^ s^−1^ and the degree of conversion of acrylate groups from 20% to 50% were observed. The effects of photoinitiator structures on the initiating ability and spectroscopic properties of sensitizers are described in this article.

## Abstract 2

The ability of two-component dyeing photoinitiating systems for the radical polymerization of 1,6-hexanediol diacrylate (HDDA) and 2-ethyl-2-(hydroxymethyl)-1,3-propanediol triacrylate (TMPTA) is presented.

## Graphical abstract

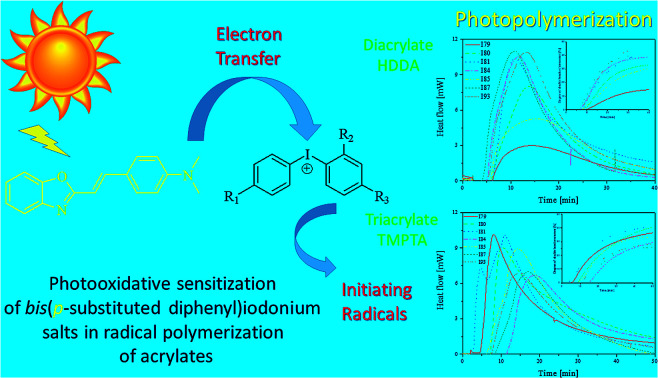

## 1. Introduction

Photopolymerization is one of the popular technologies used for the production of various types of polymer materials, which are used in various fields. In recent years, interest towards the photoinitiated polymerization has rapidly increased due to many advantages, such as low energy consumption, the possibility of using non-solvent composition and efficiency.^1,2^ This process is suitable for curing of dental fillings, fabrication of paints, coatings, lacquers, 3D objects and many others.^3^ Materials obtained by free radical polymerization (FRP) enable their further practical applications.

It is expected that these materials will be relatively cheap, compatible and have not undergone any negative changes upon the exposure to radiation and high temperatures.^4,5^

The photoinitiating system (PIS) plays a key role in FRP. For this reason, the design and development of high-performance systems, which allow the high values of kinetic parameters of the process, seem extremely important. Due to the harmful effects of ultraviolet radiation on the human body, scientists are still looking for new initiating systems for polymerization in the visible region of the spectrum.^5,6^ Free radical polymerization involves three steps: initiation, growth of the polymer chain (propagation) and termination.^7^ Usually, the initiating step of polymerization requires the application of an appropriate photoinitiator. The introduction of a dye as an absorber of light (photosensitizer) to the system shifts the sensitivity of PIS towards longer wavelengths. This molecule after absorption reaches a higher energy state (excited single or triplet state) and then decomposes or reacts with another molecule, which leads to the formation of active radicals.^8^ In general, the PISs may be classified into three groups:

• one-component (Type I): the radicals are formed *via* a homolytic α-cleavage of bonds,

• two-component (Type II): the generation of active species is related to the process of energy, electron or hydrogen atom transfer or is based on the process of the photoinduced cleavage of bonds *via* electron transfer,

• multi-component: they are composed of three or more compounds and characterized by a more complex mechanism comprising many reactions.^9^

**Scheme 1:**
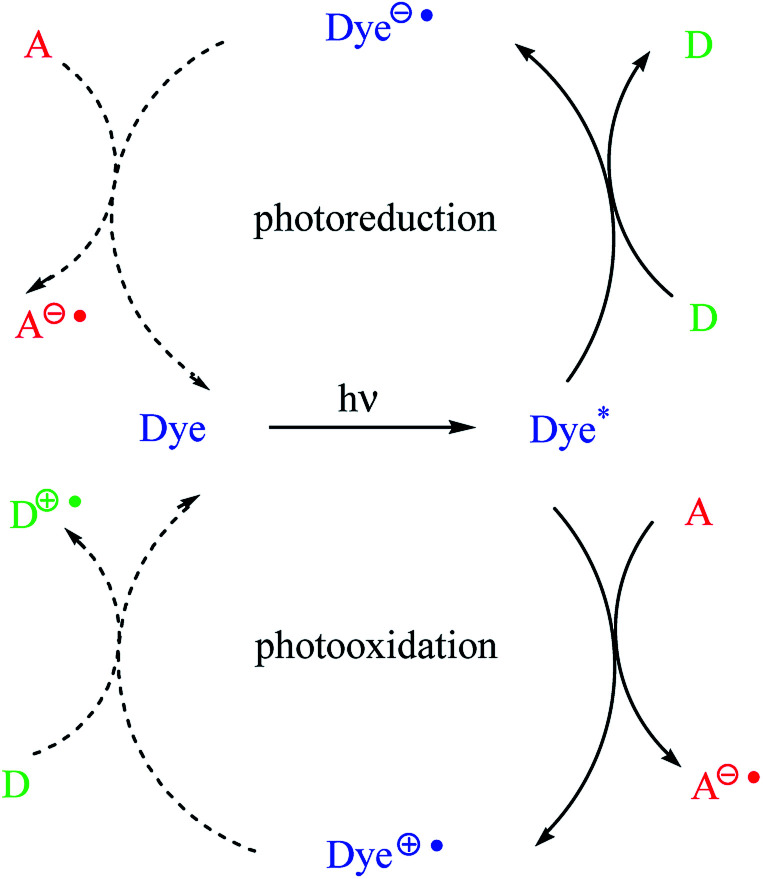
Photoinduced electron transfer in dye-sensitized photoinitiating systems.

In dyeing photoinitiating systems, there are two types of sensitization, namely, photoreducible and photooxidative, that occur *via* photoinduced electron transfer (PET) (Scheme 1). It should be noted that the dye molecules in the presence of appropriate coinitiators are capable of undergoing electron transfer reactions in the photoexcited state. The dyes may act as an electron donor or an acceptor.^7,10^

As shown in Scheme 1, the dye is a sensitizer (chromophore) molecule, for example, a hemicyanine dye. The symbols D and A correspond to electron donor (for example, alkyltriphenyl borate salt) and electron acceptor (for example, diphenyliodonium salt) molecules, respectively.

There are many photoinitiating systems based on dye molecules, as described in the literature. The synthetic chromophores, such as xanthenic dyes,^11,12^ camphorquinone,^13,14^ ketocoumarin derivatives,^15^ pyrromethenes,^16^ polymethines,^17^ and others,^18–26^ are used as absorbing species. Another example of sensitizers are neutral hemicyanine dyes. Benzothiazole-, benzoxazole- and α-napthiazole-based hemicyanines are used with different co-initiators for the initiation of the radical polymerization of triacrylates under irradiation using a 360 nm light source.^27^ Those chromophores were paired with thiophenoxyacetic acid, phenoxyacetic acid, *N*-phenylglycine, tetramethylammonium butyltriphenylborate, *N*,*N*′-dimethoxybipyridinium tetrafluoroborate, and *N*,*N*′-diethoxybipyridinium tetrafluoroborate. We did not find any information about using them with diphenyliodonium salts. In the past decades, we can find some examples for the application of diphenyliodonium salts for the initiation of photopolymerization. For example, Xiao *et al.* described the photoinitiating abilities of two- and three-component systems for the radical polymerization of TMPTA, containing naphthalimide derivatives as sensitizers, and diphenyliodonium hexafluorophosphate, *N*-vinylcarbazole, *N*-methyldiethanolamine and 2,4,6-tris(trichloromethyl)-1,3,5-triazine as coinitiators. High values of the rate of polymerization and the degree of monomer conversion above 50% were achieved.^28^

In 2019, Chen *et al.* presented new Type II photoinitiators based on benzophenone and thioxanthone, exhibiting low migration in photocuring systems. It was shown that with the increase in the light intensity from 10 mW cm^−2^ to 50 mW cm^−2^, the degree of monomer conversion increased from 56% to 89%.^29^

Lalevée *et al.*, in 2019,^30^ proposed two coumarins as high-performance visible light photoinitiators in the presence of an iodonium salt or with an amine, for both the free radical polymerization (FRP) of (meth)acrylates and the cationic polymerization (CP) of epoxides upon visible light exposure using an LED at 405 nm. Coumarin-based systems are high-performance photoinitiators that may be used for new photosensitive 3D printing resins.

The aryliodonium ylides described by the same authors^31^ are new interesting and efficient additives for the photoinitiators of radical polymerization composed of camphorquinone and amines.

In 2019, the iodonium salt as an acceptor and indoles as donors are described as stable dual thermal and photochemical free radical polymerization (FRP) initiators for benchmarked methacrylates.^32^

Due to the lack of reports on neutral hemicyanine dye/iodonium salt pairs, we decided to develop our study on dyeing photoinitiating systems acting under visible light, which are composed of neutral hemicyanine and diphenyliodonium salts. For this purpose, we have synthesized benzothiazole-based hemicyanine dyes as a blue-light-sensitive sensitizer and used several iodonium salts containing the electron-donation or electron-withdrawing groups for the polymerization of di- and triacrylates.

## 2. Experimental

### 2.1. Materials

The substrates required for the synthesis of sensitizer, *p*-(*N*,*N*-dimethylamino)benzaldehyde, 2-methylbenzoxazole and boric acid; monomers, 1,6-hexanediol diacrylate (HDDA) and 2-ethyl-2-(hydroxymethyl)-1,3-propanediol triacrylate (TMPTA); 1-methyl-2-pyrrolidinone (MP) and spectroscopic grade solvents were purchased from Sigma-Aldrich (Poland) and used without further purification. The compounds used as coinitiators in the polymerization of acrylates are diphenyliodonium chloride (I1), diphenyliodonium hexafluorophosphate (I2), (4-methoxyphenyl)-phenyliodonium *p*-toluenesulfonate (I77), (4-methoxyphenyl)-(4-methylphenyl)iodonium *p*-toluenesulfonate (I78), (4-methoxyphenyl)-(4-cyanophenyl)iodonium *p*-toluenesulfonate (I79), (4-chlorophenyl)-(4-methoxyphenyl)iodonium *p*-toluenesulfonate (I80), (4-methoxyphenyl)-(4-nitrophenyl)iodonium *p*-toluenesulfonate (I81), (3-methoxyphenyl)-(4-methoxyphenyl)iodonium *p*-toluenesulfonate (I83), (4-bromophenyl)-(4-methoxyphenyl)iodonium *p*-toluenesulfonate (I84), (4-trifluoromethylphenyl)-(4-methoxyphenyl)iodonium *p*-toluenesulfonate (I85), (2-trifluoromethylphenyl) (4-methoxyphenyl) iodonium *p*-toluenesulfonate (I86), (3-trifluoromethylphenyl)-(4-methoxyphenyl)iodonium *p*-toluenesulfonate (I87), bis(4-methoxyphenyl)iodonium *p*-toluenesulfonate (I90), (4-*tert*-butylphenyl)-(4-methoxyphenyl)iodonium *p*-toluenesulfonate (I92) and (4-fluorophenyl)-(4-methoxyphenyl)iodonium *p*-toluenesulfonate (I93). The iodonium salts: diphenyliodonium chloride (I1) and diphenyliodonium hexafluorophosphate (I2) were purchased from Sigma-Aldrich (Poland), and the others were synthesized by PhD J. Ortyl from Cracow University of Technology, as described in the literature.^33^ All substrates and solvents necessary for the preparation of coinitiators were purchased form Sigma-Aldrich (Poland) and used without further purification.

### 2.2. Spectroscopic measurements

Absorption spectra were recorded at ambient temperature using an Agilent Technologies UV-Vis Cary 60 spectrophotometer, and emission spectra were recorded using a Hitachi F-7000 spectrofluorometer. The measurements were performed for 1.0 × 10^−5^ M solutions of dye in the following solvents: diethyl ether (Et_2_O), tetrahydrofuran (THF), acetone, ethanol (EtOH), methanol (MeOH), 1-methyl-2-pyrrolidinone (MP), *N*,*N*-dimethylformamide (DMF), acetonitrile (MeCN) and dimethylsulfoxide (DMSO).

The fluorescence quantum yield of dyes in different solvents was determined by comparison with the solution of coumarin 1 in ethanol serving as the reference (*λ*_ex_ = 366 nm, *Φ*_ref_ = 0.64). The fluorescence spectra of dilute dye solution (*A* = 0.1) were registered by excitation at the maximum band of reference's absorption. This parameter was estimated using eqn (1):^34^1
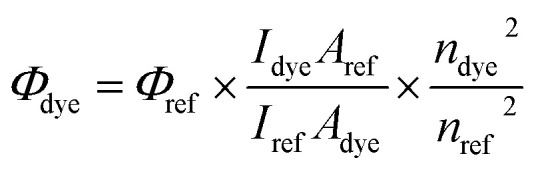
where *Φ*_dye_ is the fluorescence quantum yield of dye, *Φ*_ref_ is the fluorescence quantum yield of reference, *I*_dye_ and *I*_ref_ are the integrated emission intensities of the dye and reference, *A*_dye_ and *A*_ref_ are absorbances of the dye and reference at the excitation wavelength, *n*_dye_ and *n*_ref_ are the refractive indexes of solvents used for the dye and reference, respectively.

### 2.3. Polymerization measurements

The kinetic parameters of the free radical polymerization of multifunctional acrylate monomers were determined using a Differential Scanning Calorimeter TA DSC Q2000 Instrument equipped with a high-pressure mercury lamp (Photo-DSC). The heat evolved during reaction was registered for radiation range 300–500 nm and at a constant intensity of 30 mW cm^−2^. The measurements were performed at a sampling interval of 0.05 s per point in isothermal conditions under a nitrogen flow of 50 mL min^−1^. Due to the poor solubility of the dye in monomers, 1-methyl-2-pyrrolidinone was used as the solvent. The polymerization mixture was composed of 1.8 mL of the monomer, 0.2 mL of 1-methyl-2-pyrrolidinone, the sensitizer and an appropriate coinitiator. The concentration of photoinitiators was 5 × 10^−3^ M. The polymerizing solution without a coinitiator was used as the reference sample. The degree of conversion (*C*_%_) is directly proportional to the number of reactive groups (double bonds) in the monomer molecule. This parameter was calculated using eqn (2):2
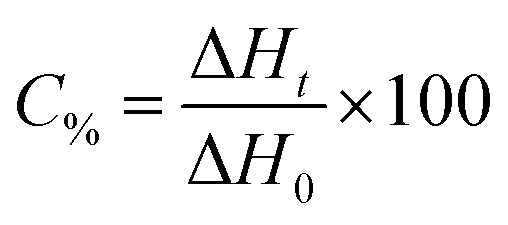
where Δ*H*_*t*_ is the reaction heat evolved at time *t* and Δ*H*_0_ is the theoretical heat for the complete degree of conversion (for acrylates: Δ*H*_0_ = 78.0 kJ mol^−1^).

The rate of polymerization (*R*_p_) is derived from the amount of heat released during the process, which is expressed by eqn (3):3
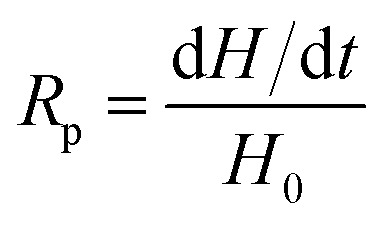
where d*H*/d*t* is a heat flow in the polymerization reaction.

Moreover, the overall ability to the initiation reaction was also calculated using eqn (4):4
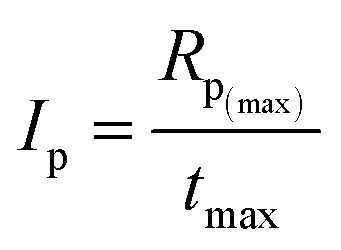
where *I*_p_ is the photoinitiation index, *R*_p_(max)__ is the maximum rate of polymerization and *t*_max_ is the time required for the maximum rate of heat release.

## 3. Results and discussion

The structures of all compounds used as components in the photoinitiating systems for polymerization experiments are depicted in Table 1.

**Table 1:** The structures and abbreviations of sensitizer, monomers and coinitiators

Sensitizer			
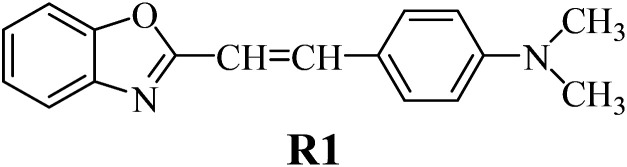			
Monomers			
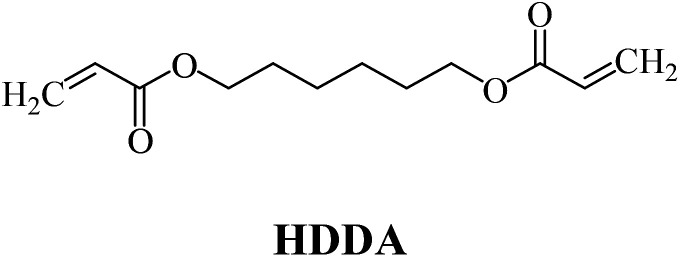		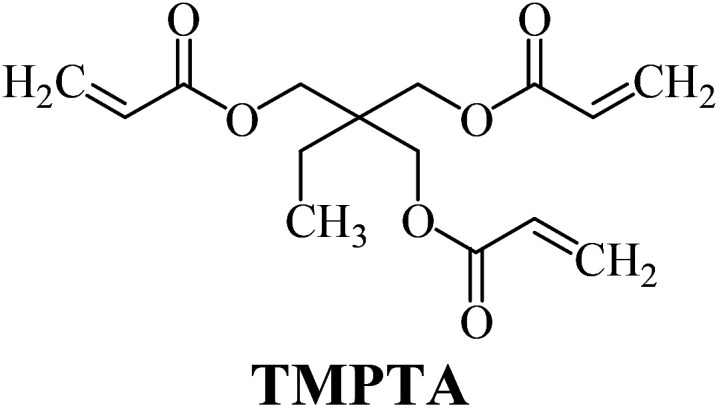	
Coinitiators			
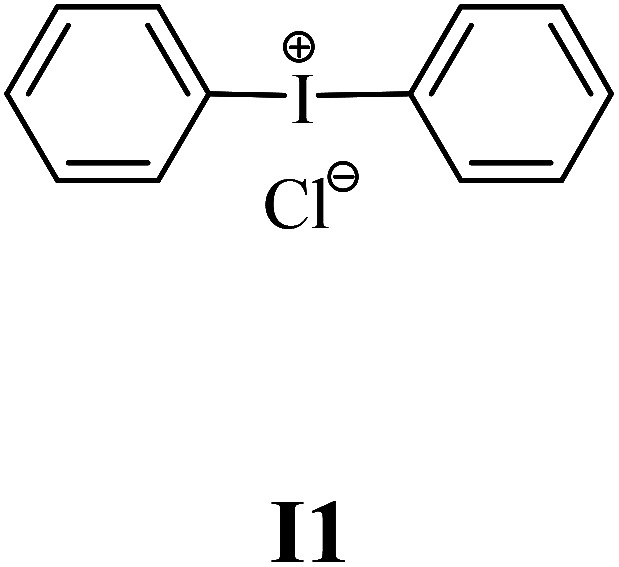	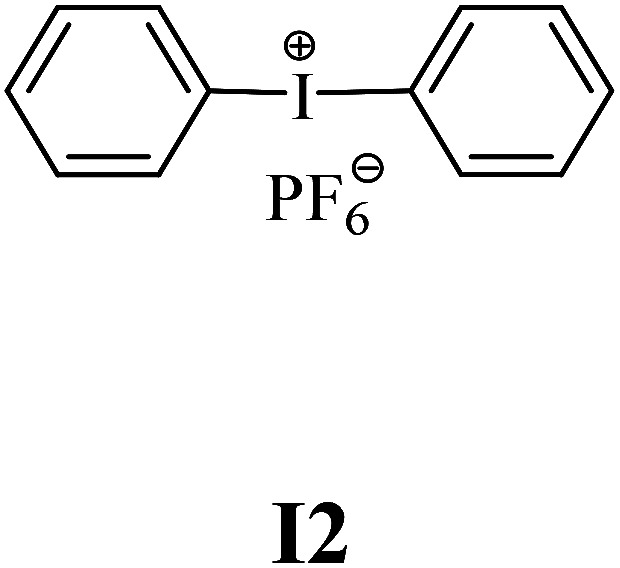	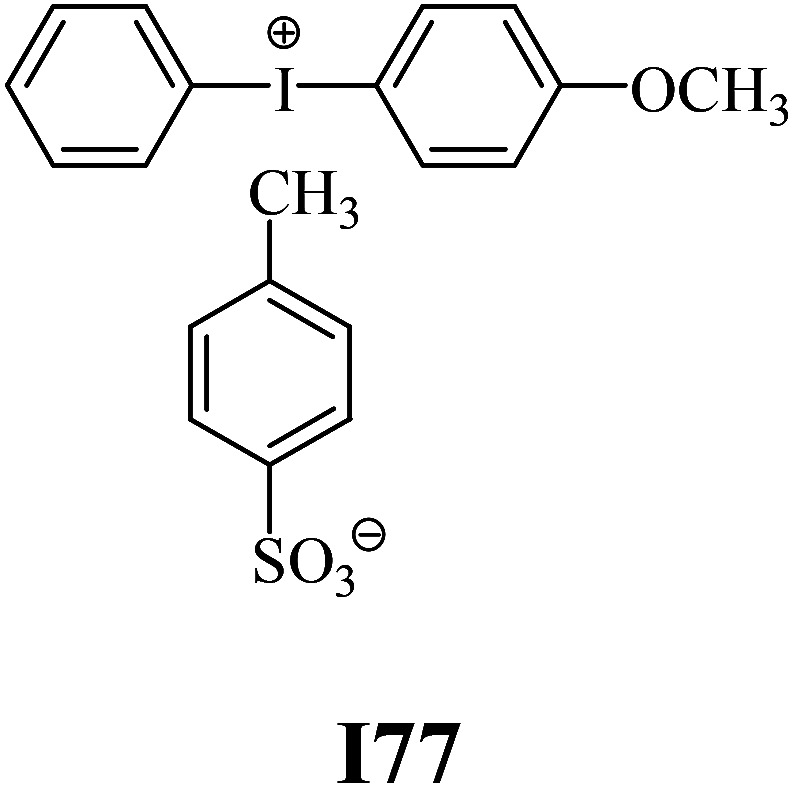	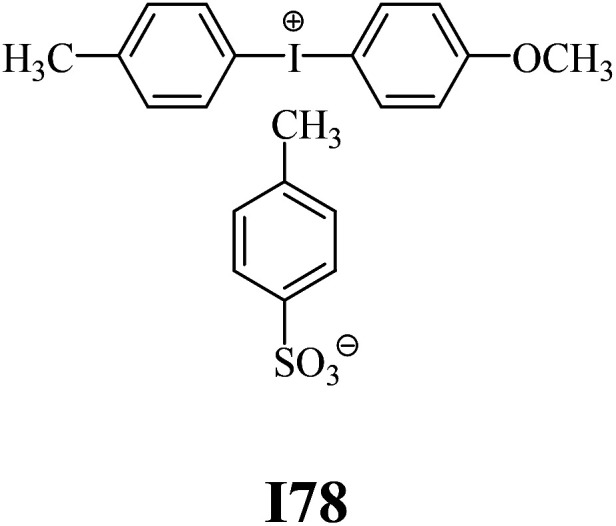
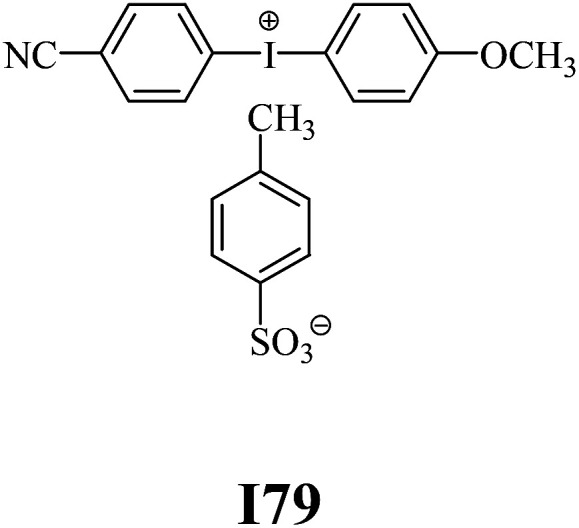	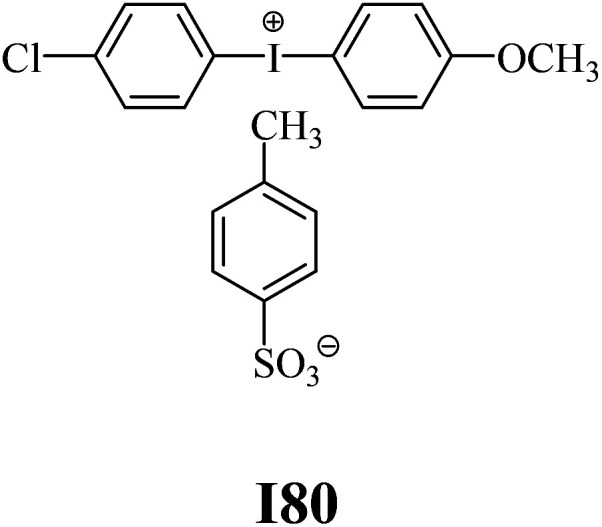	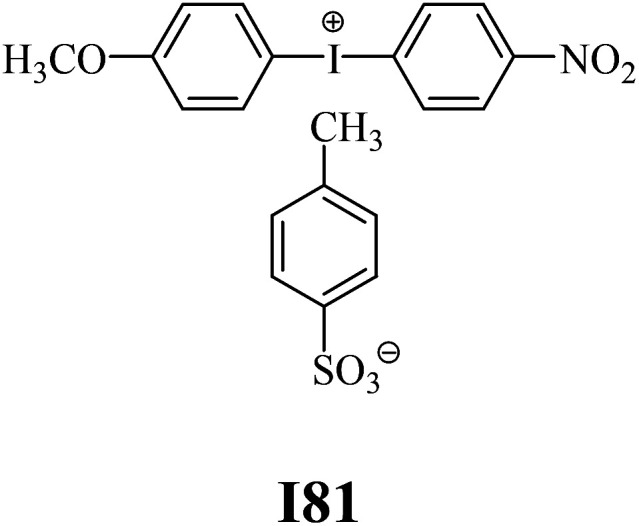	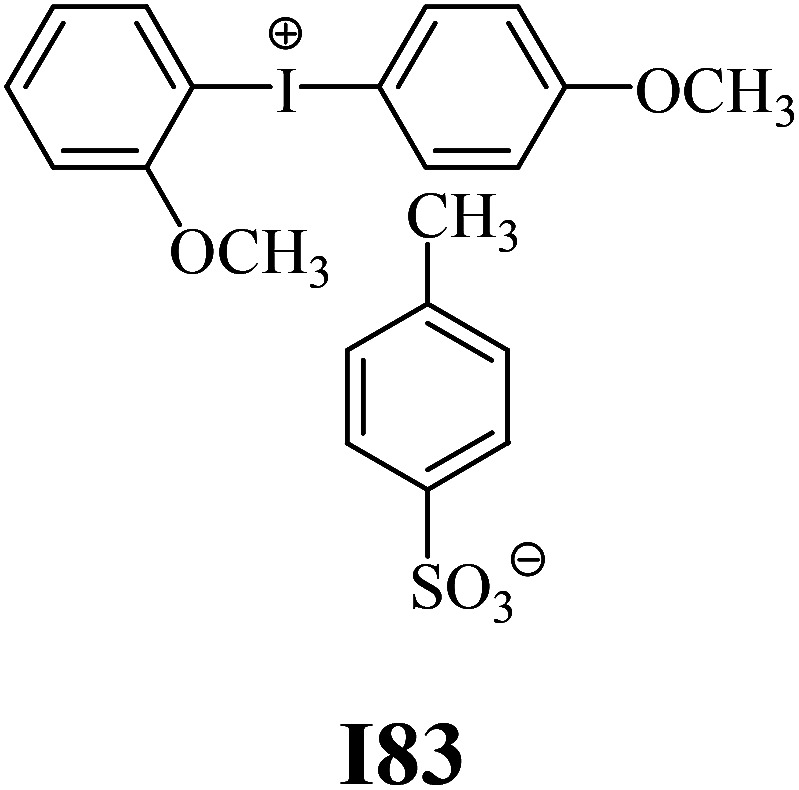
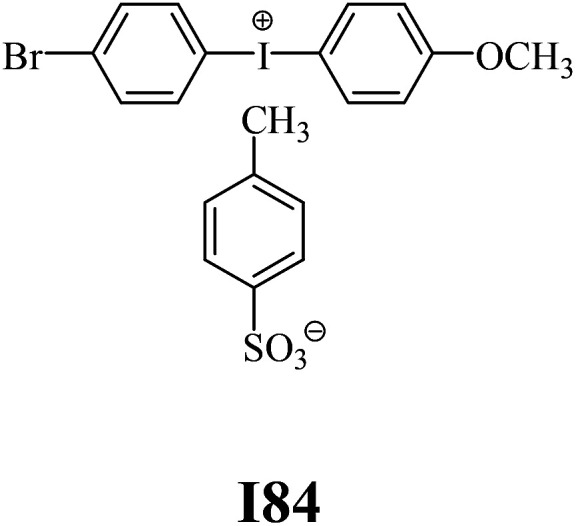	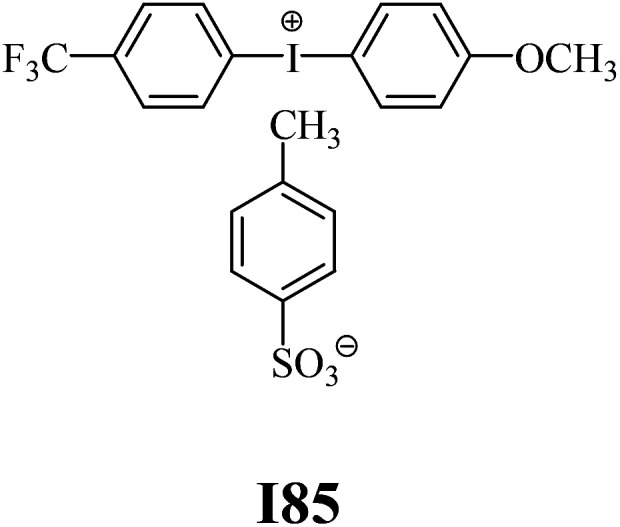	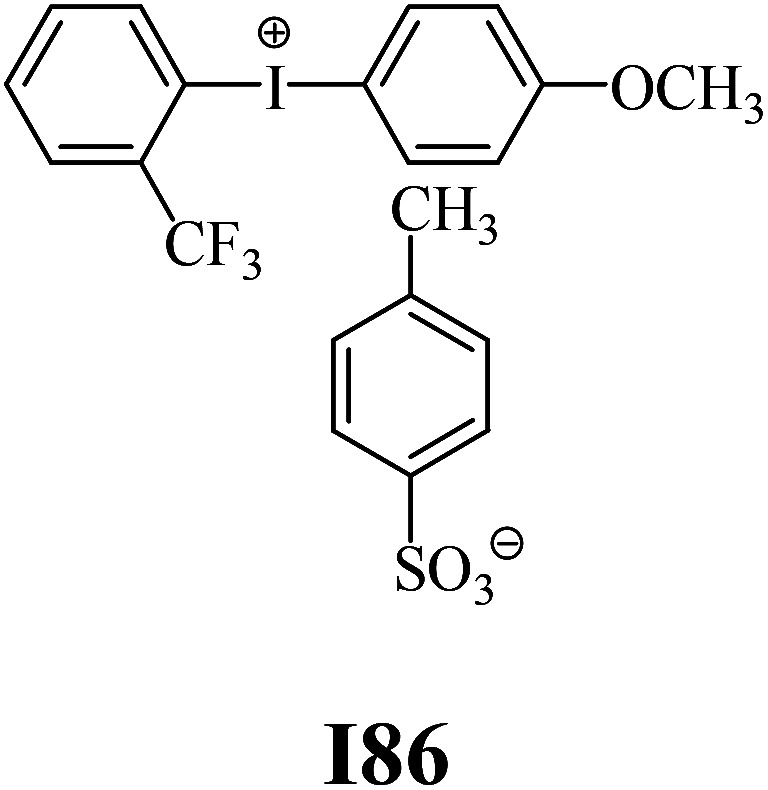	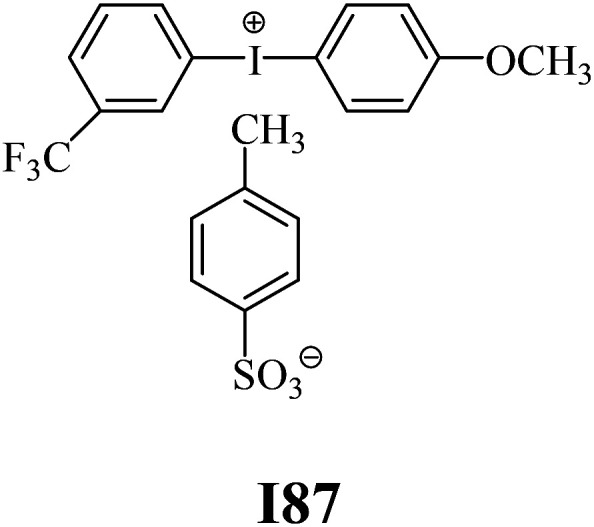
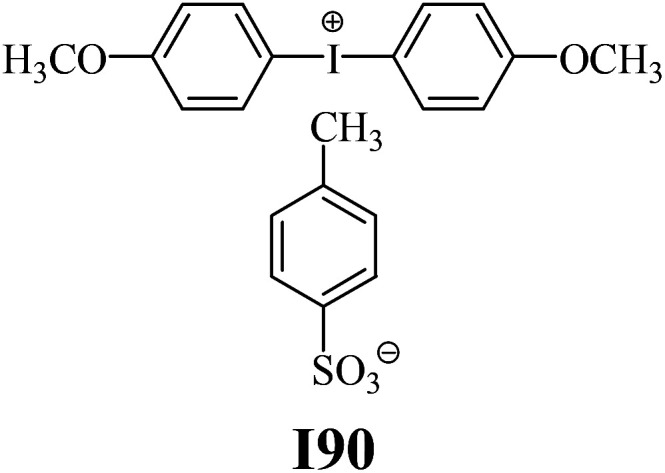	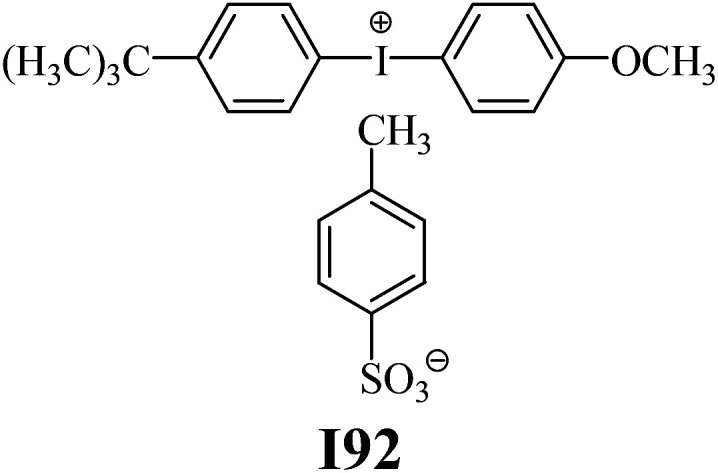		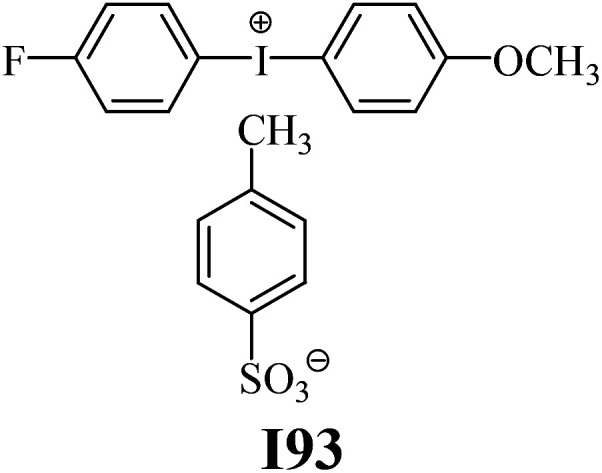

The synthetic dye 2-(*p-N*,*N*-dimethylaminostyryl)benzoxazole (R1) used as the sensitizer in the photoinitiating systems belongs to hemicyanines. The functional dyes belonging to this group possess a characteristic donor–π–acceptor (D–π–A) structure and show specific, useful properties, such as high extinction coefficients,^35^ wide range of absorption and emission radiation, good affinity for biomolecules,^36^ and good chemical activity.^37^ For recent years, the photophysical properties of cyanine dyes were intensively studied due to their applications in different areas, such as biological imaging,^38^ molecular electronics,^39^ nonlinear optics,^40^ and textile industry.^41^

There are many commercially available coinitiators, initiating both radical and cationic polymerization.^42^ The iodonium salts belong to the most commonly used coinitiators in the polymerization process.^43–45^ In our study, we present the application of series of *p*-substituted diphenyliodonium salts acting as an electron acceptor in PISs. The spectroscopic properties of the benzoxazole derivative R1 and the possibility of its application as a sensitizer in photoinitiating systems are also presented.

### 3.1. Synthesis of sensitizer

**Scheme 2:**
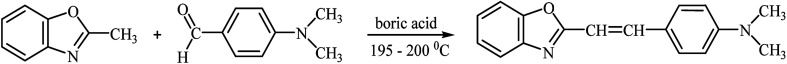
Synthesis of 2-(*p-N*,*N*-dimethylaminostyryl)benzoxazole.

2-(*p-N*,*N*-Dimethylaminostyryl)benzoxazole was synthesized by the reaction of equimolar amounts of *p*-(*N*,*N*-dimethylamino)benzaldehyde and 2-methylbenzoxazole in the presence of boric acid (0.05 g per 0.01 M of aldehyde), as shown in Scheme 2.

The mixture was heated at 195–200 °C for 5 h. The obtained compound (yellow solid) was recrystallized from ethanol and dried at ambient temperature.^46^

The structure of dye was confirmed by nuclear magnetic resonance spectroscopy. ^1^H NMR and ^13^C NMR spectra were recorded in CDCl_3_-*d*_1_ using an Ascend III spectrometer, operating at 400 MHz, Bruker (USA). Chemical shifts (*δ*) are reported in ppm relative to the internal standard (TMS) and coupling constants (*J*) expressed in Hz. The Böethius apparatus (PGH Rundfunk, Fernsehen Niederdorf KR, Stollberg/E) was used to determine the melting point (mp) of this compound.

^1^H NMR (CDCl_3_-*d*_1_), *δ* (ppm): 3.03 (s, 6H, –CH_3_); 6.71–6.73 (d, 2H, Ar); 6.83–6.87 (d, *J* = 16.2 Hz, 1H, –CH=); 7.28–7.32 (m, 2H, Ar); 7.47–7.51 (m, 3H, Ar); 7.66–7.68 (m, 2H, Ar); 7.71–7.75 (d, *J* = 16.2 Hz, 1H, –CH=).

^13^C NMR (CDCl_3_-*d*_1_), *δ* (ppm): 40.24 (–CH_3_); 108.63, 110.04–110.28, 112.11, 119.35, 124.23–124.47 (Ar); 129.13 (–CH=); 139.96, 142.47, 150.37, 151.37 (Ar).

Yield: 0.72 g, 16.81%, mp 164–167 °C.

### 3.2. Spectroscopic studies

The data characterizing the spectroscopic properties of the synthesized dye are presented in Table 2.

**Table 2:** Spectroscopic data of 2-(*p-N*,*N*-dimethylaminostyryl)benzoxazole in solvents with different polarities

Parameter	Solvent								
	Et_2_O	THF	Acetone	EtOH	MeOH	MP	DMF	MeCN	DMSO
*λ*_ab_ [nm]	380	389	388	391	391	399	393	387	398
FWHM_ab_ [cm^−1^]	4230	4408	4038	4976	5129	4642	4748	4699	4740
*ε* [10^4^ M^−1^ cm^−1^]	3.42	4.39	4.29	4.08	3.70	3.61	3.12	3.78	2.82
*λ*_fl_ [nm]	455	472	479	486	496	498	496	486	500
FWHM_fl_ [cm^−1^]	3744	3401	3182	3250	3516	3116	3116	3113	3303
Stokes shift [cm^−1^]	4338	4521	4896	4999	5414	4982	5284	5264	5126
*Φ*_dye_ [×10^3^]	4.3	7.0	6.3	14.4	6.5	15.9	11.9	7.5	12.4
*E*_00_ [eV]	2.97	2.88	2.84	2.82	2.79	2.77	2.79	2.82	2.76

In Table 2, the symbols *λ*_ab_, FWHM_ab_, *ε*, *λ*_fl_, FWHM_fl_, *Φ*_dye_ and *E*_00_ are defined as the maximum of absorption band, full width at half maximum of absorption, molar extinction coefficient, maximum of fluorescence band full width at half maximum of emission, fluorescence quantum yield and excitation energy, respectively.

**Fig. 1:**
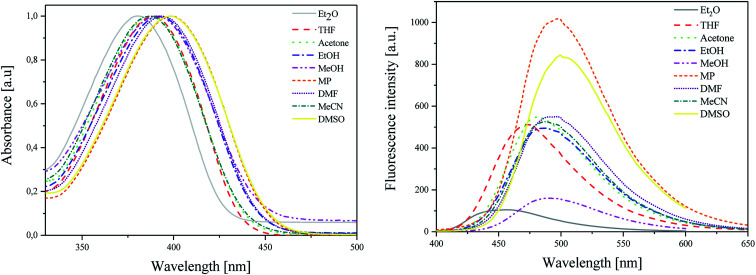
(L) Absorption and (R) emission spectra of 2-(*p-N*,*N*-dimethylaminostyryl)benzoxazole in different polarity solvents, recorded at room temperature.

As shown in Fig. 1 (L), the dye under study has a single pronounced absorption band with maximum (*λ*_ab_) located from 380 nm to 399 nm. This band is attributed to the π → π* transition.^38^ The position of the band of absorption depends on the polarity of the solvent. When the polarity increases, the absorption band shifts towards a higher wavelength (batochromic shift) from non-polar diethyl ether (Et_2_O) to polar aprotic dimethylsulfoxide (DMSO). The molar extinction coefficients (*ε*) of dye achieve relatively high values. This parameter ranges from 2.82 × 10^4^ M^−1^ cm^−1^ to 4.39 × 10^4^ M^−1^ cm^−1^. The value of Stokes shifts (Δ*ν*_max_) is equal to *ca.* 5000 cm^−1^.

The emission spectra (Fig. 1 (R)) are broad with the single maximum of fluorescence (*λ*_fl_) at about 455–500 nm. In this case, the maximum of fluorescence shifts also towards higher wavelength values as the polarity of solvent increases. The hemicyanine dye displays low values of fluorescence quantum yields of about 1 × 10^−3^.

The values of excited-state energy level (*E*_00_) increase as the polarity of solvent decreases and are in the range from 2.76 eV to 2.97 eV.

**Fig. 2:**
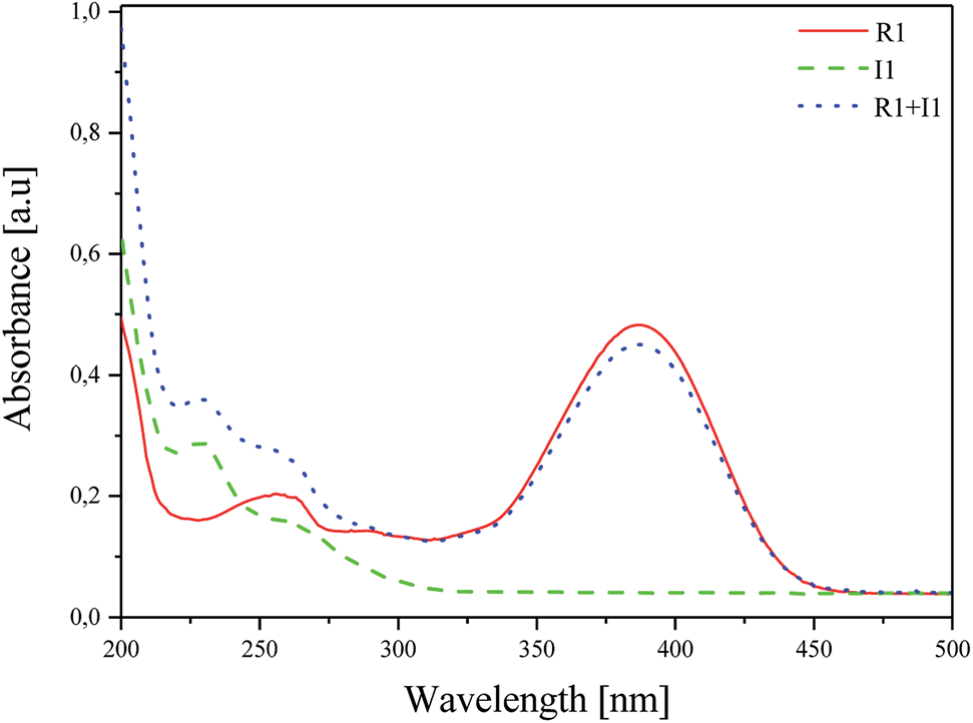
Absorption spectra of R1, I1 and their mixture R1/I1 recorded at room temperature in acetonitrile as a solvent.

The effect of the presence of coinitiators on the absorption properties of the sensitizer was studied, and the results obtained are shown in Fig. 2.^47^ For this purpose, the absorption spectra of R1 dye, iodonium salt I1 and the mixture of dye/iodonium salt in acetonitrile were recorded. The concentration was 1 × 10^−5^ M and 2 × 10^−5^ M for the dye and coinitiator, respectively.

As shown in Fig. 2, there are two bands of absorption: (i) iodonium salt in the UV region with *λ*_ab_ = 229 nm and (ii) hemicyanine dye in the Vis region with *λ*_ab_ = 386 nm. The presence of coinitiator does not have any influence on the shape and position of the absorption band of the sensitizer. The overlap of the absorption region of iodonium salt I1 and the emission of a light source is not observed. Therefore, it can be concluded that the sensitizer absorbs only the radiation emitted by the light source used, *e.g.* 300–500 nm.

### 3.3. Kinetics of the polymerization of acrylates

The influence of the combinations of sensitizer R1 and various structure diphenyliodonium salts on the kinetics parameters of the polymerization process was estimated by differential scanning calorimetry technique.

As shown in Table 1, the coinitiators used in the photopolymerization experiment differ in the type of a substituent attached to the phenyl ring in the *para* position. In general, these compounds may be divided into three groups:

• coinitiators without substituents in the *p*-position: I1, I2.

• coinitiators with an activating substituent: I77, I78, I83, I86, I90, I92.

• coinitiators with a deactivating substituent: I79, I80, I81, I84, I85, I87, I93.

It was found that the neutral hemicyanine dye R1 in the presence of diphenyliodonium salts is an efficient photoinitiator of HDDA and TMPTA polymerization (Tables 3 and 4).

Depending on the type of the coinitiator used, different values of such parameters as the heat emitted during the polymerization reaction that were directly related to its rate (*R*_p_) as well as the degree of conversion of acrylate groups (*C*_%_) for both monomers were observed.

The data presented below clearly indicated that the efficiency of the photoinitiating system depends on both the type of the coinitiator and the monomer used in polymerization experiments.

**Fig. 3:**
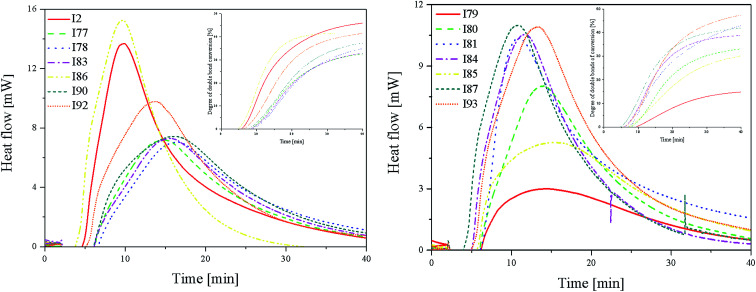
The kinetic curves recorded during the radical polymerization of HDDA initiated by 2-(*p-N*,*N*-dimethylaminostyryl)benzoxazole in the presence of coinitiators (L), with an activating substituent in the *p*-position, and (R) with a deactivating substituent in the *p*-position. Inset: time-conversion curves of the polymerization reaction in the presence of different radical sources (marked in figure).

Fig. 3 (L) shows that in the case of systems consisting of coinitiators with an activating substituent, the highest amount of heat released during the polymerization reaction was observed for the R1/I86 combination. This system exhibited the highest polymerization rate of 7.37 × 10^−4^ s^−1^. The values of the degree of monomer conversion were in the range from 33.50% to 46.49%. Furthermore, in this case, 2-methylbenzoxazole derivative in the presence of diphenyliodonium chloride (I1) did not initiate HDDA polymerization.

Among the systems R1 dye/coinitiator with the deactivating substituent (Fig. 3 (R)), the most effective was (4-methoxyphenyl)-(4-nitrophenyl)iodonium *p*-toluenesulfonate (I81). The polymerization rate was 5.0 × 10^−4^ s^−1^, while the monomer conversion exceeded 50%. The combination of R1/I79 was the least effective system to initiating the polymerization process.

**Table 3:** Kinetic parameters of the radical polymerization of HDDA initiated by systems under study

Coinitiator with electron-donating substituents	*R*_p_(max)__ [×10^−4^ s^−1^]	Monomer conversion [%]	*t*_max_ [min]	*I*_p_ [10^−7^ s^−2^]	Coinitiator with electron-withdrawing substituents	*R*_p_(max)__ [×10^−4^ s^−1^]	Monomer conversion [%]	*t*_max_ [min]	*I*_p_ [10^−7^ s^−2^]
I1^a^	—	—	—	—	I79	1.45	16.22	14.27	1.69
I2^a^	6.61	46.49	9.87	11.16	I80	3.88	33.71	14.02	4.61
I77	3.48	33.50	14.67	3.95	I81	5.01	51.21	11.02	7.58
I78	3.40	37.45	16.42	3.45	I84	5.10	39.75	11.76	7.23
I83	3.53	34.06	15.55	3.78	I85	2.54	33.38	15.46	2.74
I86	7.37	41.33	9.72	12.64	I87	5.31	43.07	10.85	8.16
I90	3.59	41.26	16.06	3.73	I93	5.28	50.17	13.31	6.61
I92	4.73	42.83	13.70	5.75					

a Coinitiators without substituents in the *p*-position.

**Fig. 4:**
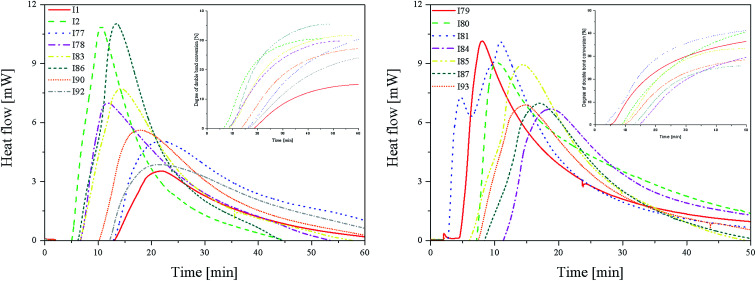
The kinetic curves recorded during the radical polymerization of TMPTA initiated by 2-(*p-N*,*N*-dimethylaminostyryl)benzoxazole in the presence of coinitiators (L): with an activating substituent in the *p*-position and (R): with a deactivating substituent in the *p*-position. Inset: time-conversion curves of polymerization reaction in the presence of different radical sources (marked in figure).

From the kinetics curves (Fig. 4) and data summarized in Table 4, it can be concluded that the highest final monomer conversion was obtained for the systems, containing coinitiators with electron-withdrawing substituents. The maximum rate of polymerization oscillated about 3–4 × 10^−4^ s^−1^ and the degree of double bond monomer conversion achieved values from 30% to even 40%. Similar to the polymerization of HDDA, the sensitizer R1 in the presence of diphenyliodonium chloride (I1) is not an effective photoinitiator of the polymerization of TMPTA. The effectiveness to the initiation of polymerization is higher for systems, containing coinitiators with strong deactivating substituents, such as –CN and –NO_2_. For both cases, the *R*_p_ value achieved *ca.* 4.30 × 10^−4^ s^−1^.

**Table 4:** Kinetic parameters of the radical polymerization of TMPTA initiated by systems under study

Coinitiator with electron-donating substituents	*R*_p_(max)__ [×10^−4^ s^−1^]	Monomer conversion [%]	*t*_max_ [min]	*I*_p_ [10^−7^ s^−2^]	Coinitiator with electron-withdrawing substituents	*R*_p_(max)__ [×10^−4^ s^−1^]	Monomer conversion [%]	*t*_max_ [min]	*I*_p_ [10^−7^ s^−2^]
I1^a^	1.49	15.15	22.00	1.13	I79	4.28	41.63	8.10	8.81
I2^a^	4.59	30.56	10.73	7.13	I80	3.81	44.64	10.47	6.06
I77	2.13	32.13	21.75	1.63	I81	4.26	42.41	10.94	6.49
I78	2.95	29.84	11.81	4.16	I84	2.83	32.40	18.74	2.52
I83	3.26	31.71	14.24	3.82	I85	3.77	33.31	14.34	4.38
I86	4.65	35.54	13.45	5.76	I87	2.95	25.88	16.98	2.90
I90	2.37	27.36	17.79	2.22	I93	2.91	29.03	14.90	3.26
I92	1.63	24.89	21.40	1.27					

a Coinitiators without substituents in the *p*-position.

The values of photoinitiation index (*I*_p_) show that all of the compounds used as coinitiators in the polymerization process exhibit similar effectiveness to the generation of active centers. This parameter achieved values of about 10^−7^ s^−2^.

**Fig. 5:**
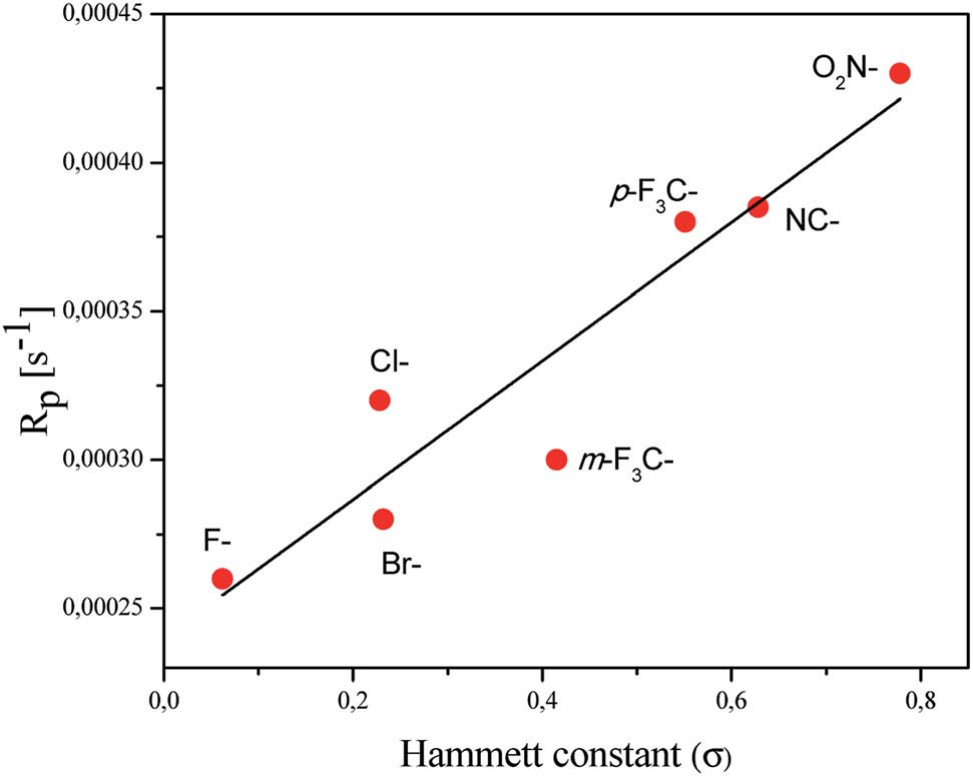
Correlation between the polymerization rate of TMPTA (*R*_p_) and the Hammett parameter (*σ*).

In order to explain the relation between the structure of the coinitiator used and the ability of the system to initiate free radical polymerization, the dependence of the polymerization rate on the Hammett substituent constants is presented (Fig. 5).

A linear correlation of the *R*_p_ values with the Hammett constants *σ* for the deactivating substituents was observed. The correlation coefficient *R*^2^ was determined to be 0.89. The system containing a coinitiator with a deactivating substituent characterized by a higher value parameter of *σ* effectively initiated the polymerization of TMPTA. Accordingly, the highest *R*_p_ value was reached for the combination of 2-(4-*N*,*N*-dimethylaminostyryl)benzoxazole/(4-methoxyphenyl)-(4-nitrophenyl)iodonium *p*-toluenesulfonate (R1/I81).

In general, in dye-sensitized photoinitiating systems, the photoinduced electron transfer process plays a key role in the formation of free radicals.

In this endothermal process, the absorbed light quantum initiates an electron transfer from the donor to the acceptor.^5,9^ In order to confirm that in photoinitiating systems under study, an electron transfer is possible from a thermodynamic point of view, the change of free energy for electron transfer process was calculated on the basis of Rehm–Weller equation (eqn (5)).^48^ The obtained data are summarized in Table 5.

5 Δ*G*_et_ = *E*_ox_ − *E*_red_ − *E*_00_ + *C*

where: Δ*G*_et_ is the free energy change for an electron transfer reaction, *E*_ox_ is the oxidation potential of an electron donor, *E*_red_ is the reduction potential of an electron acceptor, *E*_00_ is the excited-state energy level, *C* is a constant depending on the degree of charge separation (negligible value for polar solvents).

**Table 5:** Thermodynamic data for dye and iodonium salts^20^

Hemicyanine dye R1	*E*_ox_ [eV]				
	0.925				
Electron acceptor	*E*_red_ [eV]	Δ*G*_et_^a^ [eV]	Electron acceptor	*E*_red_ [eV]	Δ*G*_et_^a^ [eV]
I1	−0.494	−1.401	I84	−0.175	−1.720
I2	−1.000	−0.895	I85	−0.106	−1.789
I77	−0.206	−1.689	I86	−0.260	−1.635
I78	−0.395	−1.500	I87	−0.254	−1.641
I79	−0.200	−1.695	I90	−0.342	−1.553
I80	0.292	−1.603	I92	−0.310	−1.585
I81	−0.554	−1.341	I93	−0.302	−1.593
I83	−1.151	−0.744			

a Value of *E*_00_ for R1 in acetonitrile: *E*_00_ = 2.82 eV (see Table 2).

Analyzing the data presented in Table 5, it can be seen that the free energy change for electron transfer process ranges from −0.744 eV to −1.789 eV. The negative values of Δ*G*_et_ demonstrated that as a result of an electron transfer reaction between the dye and the coinitiator for all studying photoinitiating systems the radicals initiating polymerization are formed. Moreover, the iodonium salts are an electron acceptor and the dye acts as an electron donor.

**Scheme 3:**
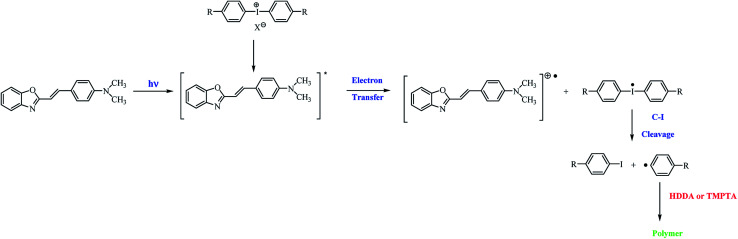
General mechanism of the formation of initiating radicals for the photopolymerization process.

Based on the nanosecond laser flash photolysis results described earlier, the mechanism of the formation of radicals in two-component photoinitiating systems composed of hemicyanine dye and iodonium salt has been proposed (Scheme 3).^7^

After the irradiation of the polymerizing mixture, the excited state of the dye is formed. In the presence of iodonium salts, the deactivation of the excited state of the sensitizer *via* electron transfer from the dye molecule to the coinitiator occurs, and diphenyliodonium radical decomposes *via* the cleavage of the carbon–iodide bond. The *p*-substituted iodobenzene and *p*-substituted active phenyl radicals, which initiate the polymerization reaction, are formed.

## 4. Conclusions

The benzoxazole derivative in combination with iodonium salts is a high-performance photoinitiator for the free radical polymerization of both HDDA and TMPTA. The synthesized dye intensively absorbs radiation in the UV-Vis region; therefore, this molecule may be used as a sensitizer in PISs. The photoinitiating ability is related to the composition of photoinitiating system. It depends on both the structure of coinitiators and the type of monomers used in the experiment. The rate of polymerization increases with the increase in Hammett constants. The initiating radicals are formed *via* a photoinduced electron transfer process. The mechanism of the formation of radical sources is presented. The mechanism of the generation of active centers involves an electron transfer from the excited state of the dye to the coinitiator molecule. The obtained results allowed us to evaluate the effectiveness of new systems that can be used as photoinitiators of the free radical polymerization of acrylates in the UV-Vis region.

## Conflicts of interest

There are no conflicts to declare.

## Supplementary Material
